# The 2012 Briganti nomogram not only predicts lymph node involvement but also disease progression in surgically treated intermediate-risk prostate cancer patients with PSA <10 ng/mL, ISUP grade group 3, and clinical stage up to cT2b

**DOI:** 10.1590/S1677-5538.IBJU.2024.0003

**Published:** 2024-05-20

**Authors:** Antonio Benito Porcaro, Andrea Panunzio, Rossella Orlando, Francesca Montanaro, Alberto Baielli, Francesco Artoni, Sebastian Gallina, Alberto Bianchi, Giovanni Mazzucato, Emanuele Serafin, Giulia Marafioti Patuzzo, Alessandro Veccia, Riccardo Rizzetto, Matteo Brunelli, Filippo Migliorini, Riccardo Bertolo, Alessandro Tafuri, Maria Angela Cerruto, Alessandro Antonelli

**Affiliations:** 1 Department of Urology University of Verona Azienda Ospedaliera Universitaria Integrata Verona Italy Department of Urology, University of Verona, Azienda Ospedaliera Universitaria Integrata, Verona, Italy;; 2 Department of Urology Vito Fazzi Hospital Lecce Italy Department of Urology, Vito Fazzi Hospital, Lecce, Italy;; 3 Department of Pathology University of Verona Azienda Ospedaliera Universitaria Integrata Verona Italy Department of Pathology, University of Verona, Azienda Ospedaliera Universitaria Integrata, Verona, Italy

**Keywords:** Prostatic Neoplasms, Minimally Invasive Surgical Procedures, Lymph Node Excision

## Abstract

**Purpose:**

We assessed the prognostic impact of the 2012 Briganti nomogram on prostate cancer (PCa) progression in intermediate-risk (IR) patients presenting with PSA <10ng/mL, ISUP grade group 3, and clinical stage up to cT2b treated with robot assisted radical prostatectomy eventually associated with extended pelvic lymph node dissection.

**Materials and Methods:**

From January 2013 to December 2021, data of surgically treated IR PCa patients were retrospectively evaluated. Only patients presenting with the above-mentioned features were considered. The 2012 Briganti nomogram was assessed either as a continuous and a categorical variable (up to the median, which was detected as 6%, vs. above the median). The association with PCa progression, defined as biochemical recurrence, and/or metastatic progression, was evaluated by Cox proportional hazard regression models.

**Results:**

Overall, 147 patients were included. Compared to subjects with a nomogram score up to 6%, those presenting with a score above 6% were more likely to be younger, had larger/palpable tumors, presented with higher PSA, underwent tumor upgrading, harbored non-organ confined disease, and had positive surgical margins at final pathology. PCa progression, which occurred in 32 (21.7%) cases, was independently predicted by the 2012 Briganti nomogram both considered as a continuous (Hazard Ratio [HR]:1.04, 95% Confidence Interval [CI]:1.01-1.08;p=0.021), and a categorical variable (HR:2.32; 95%CI:1.11-4.87;p=0.026), even after adjustment for tumor upgrading.

**Conclusions:**

In IR PCa patients with PSA <10ng/mL, ISUP grade group 3, and clinical stage up to cT2b, the 2012 Briganti nomogram independently predicts PCa progression. In this challenging subset of patients, this tool can identify prognostic subgroups, independently by upgrading issues.

## INTRODUCTION

Prostate cancer (PCa) is currently classified into prognostic risk groups according to international guidelines stated by the European Association of Urology (EAU) (1), and the National Comprehensive Cancer Network (NCCN) (2). However, the two classification systems are not equivalent, especially for the intermediate-risk (IR) group, which is also the most controversial due to the inclusion of different clusters of cancer (3, 4). Accordingly, the presence of a bilateral palpable tumor that qualifies patients for the IR group by the NCCN, is consider as a factor of high-risk disease by the EAU (1, 2). Assessing favorable prognostic subgroups within the IR class may be pivotal for deciding treatment options that not impair quality of life, which is an important matter for PCa patients (5, 6). Nevertheless, unfavorable tumor grade in the surgical specimen may become a serious life-threatening drawback after radical prostatectomy, which is more frequently performed by the robot assisted approach (RARP) eventually associated with extended pelvic lymph node dissection (ePLND) (1, 2, 7, 8).

Interestingly, IR PCa patients presenting with a tumor involving one lobe (clinical stage T2b) or less, prostate-specific antigen (PSA) <10 ng/mL, and International Society of Urological Pathology (ISUP) grade group 3 represent a borderline subgroup in whom the risk of tumor upgrading (ISUP > 3) is a serious drawback due to the association with unfavorable tumor stage (extracapsular extension or seminal vesicle invasion) at final pathology, and eventually with tumor progression (9). Nevertheless, not all upgraded and upstaged patients will experience disease progression (1, 2). Accordingly, clinicians need predictors of PCa progression that are not represented by molecular biology, which is still far from daily routine, neither by multiparametric magnetic resonance imaging (mpMRI), which is not reproducible for being operator dependent (1, 2, 10).

In patients undergoing RARP, the risk of pelvic lymph node involvement (PLNI) is an important issue usually assessed by validated nomograms (11), of which the 2012 Briganti one remains the most applied in daily practice for its easy to compute and to reproduce; accordingly, ePLND is recommended when the risk is over 5% (12); nevertheless, it has not yet been evaluated as a prognostic factor after surgery.

The aim of this study was to evaluate the 2012 Briganti nomogram on prediction of PLNI as a prognostic factor of PCa progression after RARP eventually associated with ePLND in a particular subset of IR PCa patients presenting with PSA <10 ng/mL, clinical stage up to cT2b, and ISUP grade group 3. We hypothesized that in these patients the nomogram not only predicts PLNI at final pathology, but also associates with disease recurrence and/or progression.

## MATERIALS AND METHODS

### Study population

The study had Institutional Board Review approval. All patients signed informed consent for using the data. We retrospectively evaluated data of 527 IR PCa patients, according to the EAU classification (1), who underwent RARP eventually associated with ePLND at the Integrated University Hospital of Verona between January 2013 and December 2021. Only patients presenting with PSA <10 ng/mL, clinical stage up to cT2b, and ISUP grade group 3 were considered. Subjects with no available follow-up data were excluded.

Demographics, clinical, and pathological information of each patient were recorded. These included age (years), body mass index (BMI; kg/m^2^), preoperative physical status assessed through the American Society of Anesthesiologists classification system (13), PSA (ng/mL), prostate volume (PV, mL), percentage of biopsy positive cores (BPC), and tumor stage and grade assessed according to the Tumor Node Metastasis (TNM, 8th edition, 2017 version) and the ISUP systems, respectively (14, 15). Radical prostatectomy was performed by five skilled surgeons through the robot-assisted approach, eventually associated with ePLND, which included the removal of external iliac, obturator, Cloquet’s and Marcille’s lymph nodes (16, 17). Surgical specimens were evaluated by a dedicated uro-pathologist for tumor grade, stage and cancer invasion of surgical margins as already reported. Patients were followed up according to guidelines recommendation, and discussed in a multidisciplinary setting, when further treatments were eventually addressed (1, 2).

### Study design, outcomes of interest, and statistical methods

The aim of the present study was to assess the role of the 2012 Briganti nomogram as a predictor of tumor progression after RARP in IR PCa patients, according to the EAU, with PSA less than 10 ng/mL, clinical stage up to T2b, and ISUP grade group 3. The nomogram score (%) was evaluated both as a continuous, and a categorical variable (dichotomized as up to the median vs. above the median). The event of disease progression was defined as the occurrence of biochemical recurrence/persistence and/or local recurrence and/or distant metastases. The study endpoint was compared with tumor upgrading (ISUP >3) in the surgical specimen.

Descriptive statistics included frequencies and proportions for categorical variables. Medians and interquartile range (IQR) were reported for continuously coded variables. Associations between the nomogram score and clinical and pathological factors were computed by univariable and multivariable logistic regression models. The length of time between surgery and disease progression or the last follow-up was measured as time to event occurrence. Kaplan-Meier plots depicted PCa progression-free survival according to the nomogram score and tumor upgrading at final pathology. Accordingly, associations with PCa progression were assessed by multivariable Cox proportional hazards regression analysis; here, Wald’s forward method was applied, because of the multicollinearity of factors. IBM-SPSS version 26.0 (IBM Corp., Armonk, NY, USA) was used for all analyses. All tests were two-sided with p < 0.05 considered to indicate statistical significance.

## RESULTS

### Descriptive characteristics of the patient population

Overall, 147 patients were included in the study and were stratified according to the median 2012 Briganti score, which was detected as 6% (IQR: 4 -11%), as shown in Table-1. Compared to subjects with a nomogram score up to 6%, those presenting with a score above 6% were more likely to be younger (65 vs. 69 years), to have larger/palpable tumors (63.6% vs. 32.0%), and to present with higher PSA values (6.8 vs. 5.6 ng/mL). Additionally, these patients were more likely to undergo tumor upgrading (37.5% vs. 17.3%), to harbor non-organ confined disease (31.9% vs. 12.0%), and to had positive surgical margins (26.4% vs. 6.7%) at final pathology. Pelvic lymph nodes were staged in 120 (81.6%) cases, and PLNI was assessed in 7 (5.8%) cases. The median number of counted lymph nodes was 23.5 (IQR: 17 – 32), with no significant difference between groups.

**Table 1:** Descriptive statistics of the study population stratified according to the 2012 Briganti nomogram score predicting the risk of lymph node invasion (up to the median vs above the median (*), and logistic regression models of association of clinical and pathological factors with a 2012 Briganti nomogram risk score >6%.

	Overall cohort	Briganti nomogram risk score ≤ 6%	Briganti nomogram risk score > 6%	Univariable		Multivariable (*)	
	n = 147	n = 75 (51.1)	n = 72 (48.9)	OR (95% CI)	P-value	OR (95% CI)	P-value
Age (years)	66 (61 – 71)	69 (62 - 72)	65 (61 - 70)	0.94 (0.88 - 0.99)	0.026	0.91 (0.85 - 0.98)	0.016
BMI (kg/m^2)	25.6 (23.7 - 27.9)	25.9 (23.9 - 27.8)	25.6 (23.1 - 28.3)	1.01 (0.91 - 1.11)	0.9		
**ASA**							
1	10 (6.8)	5 (6.7)	5 (6.9)	Ref.	-		
2	129 (81.6)	60 (80.0)	60 (83.3)	1 (0.28 - 3.63)	1.0		
3	17 (11.6)	10 (13.3)	7 (9.8)	0.70 (0.15 - 3.37)	0.7		
PV (mL)	36.5 (29.0 - 46.0)	38.1 (30.0 - 50.0)	34.0 (28.0 - 45.0)	0.99 (0.96 - 1.01)	0.2		
PSA (ng/mL)	6.3 (5.0 - 8.0)	5.6 (4.9 - 7.3)	6.8 (5.2 - 8.5)	1.24 (1.03 - 1.45)	0.022		
BPC (%)	31.2 (20.0 - 50.0)	20.0 (14.2 - 28.5)	48.6 (33.3 - 66.1)	1.11 (1.07 - 1.15)	<0.001	1.11 (1.07 - 1.15)	<0.001
**Clinical stage**							
T1c	74 (52.1)	51 (68.0)	26 (36.1)	Ref.	-	Ref.	-
T2b	68 (47.9)	24 (32.0)	46 (63.9)	3.76 (1.90 - 7.44)	<0.001	2.99 (1.15 - 7.77)	0.024
PW (gr)	50.0 (42.0 - 60.0)	50.0 (43.0 - 63.0)	48.5 (40.2 - 60.0)	0.99 (0.96 - 1.01)	0.2		
**Tumor upgrade**							
**No (ISUP ≤3)**	107 (72.8)	62 (82.7)	45 (62.5)	Ref.	-	Ref.	-
**Yes (ISUP >3)**	40 (27.2)	13 (17.3)	27 (37.5)	2.86 (1.33 - 6.15)	0.007	3.36 (1.07 - 10.61)	0.039
**Tumor stage**							
pT2 (organ confined)	115 (78.2)	66 (88.0)	49 (68.1)	Ref.	-		
pT3 (non-organ confined)	32 (21.8)	9 (12.0)	23 (31.9)	3.44 (1.47 - 8.09)	0.005		
**Surgical margins**							
Negative (R0)	123 (83.7)	70 (93.3)	53 (73.6)	Ref.	-		
Positive (R1)	24 (16.3)	5 (6.7)	19 (26.4)	5.02 (1.76 - 14.31)	0.003		

**Abbreviations:** OR = odds ratio; CI = confidence interval; BMI = body mass index; ASA = American Society of Anesthesiologists; PV = prostate volume; PSA = prostate-specific antigen; BPC = biopsy positive cores; PW = prostate weight; ISUP = International Society of Urological Pathology.

(*) median (interquartile range): 6 (4-11)%; (**) according to Wald's forward method.

Continuous variables are reported as median (interquartile range) while categorical factors as frequencies (percentage).

### Prognostic impact of 2012 Briganti nomogram on PCa progression after RARP

Median follow-up was 74.3 (IQR: 68.2 – 80.4) months. Overall, tumor upgrading (ISUP >3) at final pathology occurred in 40 (27.2%) patients, while PCa progression occurred in 32 (21.7%) cases, of whom 13 (17.3%) belonged to the nomogram score ≤6% group, and 19 (26.4%) belonged to nomogram score >6% group.

**Figure 1:**
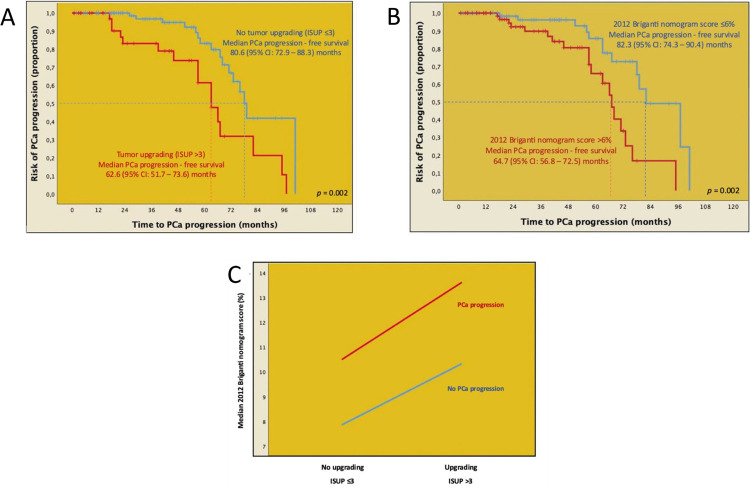
Kaplan-Meier plots depicting prostate cancer (PCa) progression – free survival according to A) tumor upgrading (ISUP grade group >3); B) the 2012 Briganti nomogram score (≤6% vs. >6%), and C) line plot illustrating the distribution of the 2012 Briganti nomogram score stratified by tumor upgrading and cancer progression in the overall cohort of 147 EAU intermediate-risk patients presenting with PSA <10 ng/mL, clinical stage up to cT2b, and ISUP grade group 3 and treated with robot assisted radical prostatectomy eventually associated with extended pelvic lymph node invasion.

Kaplan-Meier plots depicted PCa progression – free survival according to tumor upgrading (ISUP ≤3 vs. >3), and the 2012 Briganti nomogram score categorized as ≤6% vs. >6%. Specifically, median PCa progression – free survival was higher for not upgraded (80.6 months, 95%CI: 72.9 – 88.3) than for upgraded cases (62.6 months, 95%CI: 51.7 – 73.6) with the difference being statistically significant (Mantel-Cox log rank test: p = 0.002; Figure-1A). Median PCa progression - free survival was higher in patients presenting with a nomogram score up to 6% (82.3 months, 95%CI: 74.3 – 90.4) compared to patients with a score above 6% (64.7 months, 95%CI: 56.8 – 72.5) with the difference being equally statistically significant (Mantel-Cox log rank test: p = 0.002; Figure-1B).

At multivariable Cox regression analysis, that also accounted for other available clinical factors, PCa progression was independently predicted by the 2012 Briganti nomogram evaluated as a continuous variable (Hazard Ratio [HR]: 1.06; 95% Confidence Interval [CI]: 1.02 – 6.98; p = 0.001), as reported in Table-2. Accordingly, as the nomogram score increased, so patients were more likely to experience disease progression. The 2012 Briganti nomogram maintained the status of independent predictor of PCa progression even after adjustment for tumor upgrading, when evaluated both as a continuous (HR: 1.04; 95% CI: 1.01 – 1.08; p = 0.021), as well as a categorical variable (HR: 2.32; 95% CI: 1.11 – 4.87; p = 0.026).

**Table 2:** Descriptive statistics of the study population stratified according to disease progression, and Cox regression models of association of clinical factors with the risk of disease progression.

	No PCa progression n = 115 (78.2)	PCa progression n = 32 (21.7)	Univariable		Multivariable (*)	
			HR (95% CI)	*P-value*	HR (95% CI)	*P-value*
**Age (years)**	67 (61 - 71)	65 (61 - 70)	0.99 (0.94 - 0.105)	0.8		
**BMI (kg/m^^2^)**	25.7 (23.7 - 27.8)	25.1 (23.7 - 28.5)	0.99 (0.87 - 1.12)	0.8		
**PV (mL)**	37.0 (30.0 - 46.0)	33.0 (25.0 - 48.5)	0.98 (0.96 - 1.01)	0.2		
**PSA (ng/mL)**	6.3 (5.0 - 8.1)	6.3 (5.1 - 7.9)	1.13 (0.93 - 1.37)	0.2		
**BPC (%)**	28.5 (20.0 - 45.4)	47.0 (20.3 - 64.2)	1.02 (1.01 - 1.04)	0.001		
**Clinical stage**						
T1c	59 (51.3)	18 (56.3)	Ref.	-		
T2b	56 (48.7)	14 (43.7)	2.31 (1.12 - 4.76)	0.024		
**2012 Briganti nomogram score**	6.0 (4.0 – 11.0)	7.5 (4.0 - 16.5)	1.06 (1.02 - 1.09)	0.001	1.06 (1.02 - 1.09)	0.001

Abbreviations: HR = odds ratio; CI = confidence interval; BMI = body mass index; PV = prostate volume; PSA = prostate-specific antigen; BPC = biopsy positive cores. / (*) according to Wald’s forward method. Continuous variables are reported as median (interquartile range) while categorical factors as frequencies (percentage).

The distribution of the 2012 Briganti nomogram median score stratified by the occurrence of tumor upgrading and PCa progression is illustrated in Figure-1C, showing that nomogram median values were higher for progressing upgraded patients compared to those upgraded but not experiencing recurrence. Similarly, not upgraded but progressing subjects were more likely to have higher median nomogram values at clinical presentation compared to those not harboring upgrading and not experiencing progression.

## DISCUSSION

More prognostic factors predicting the natural history of PCa after primary treatments are needed. About 30-35% of patients treated with surgery will experience PCa recurrence, which will progress to fatal disease in 16.4% of cases (18-20). A large study has shown that early PSA recurrence and adverse tumor grades (ISUP >3) predicted disease progression, as well as cancer specific mortality after surgery or radiation therapy; nevertheless, these investigators outlined that accuracy could be improved by nomograms including simultaneously multiple factors (21). So far, adverse tumor grades leading to early biochemical recurrence, which will progress to metastatic and fatal disease, as well. The pivotal importance of adverse tumor grade as a prognostic factor of PCa natural history has also been confirmed by the Cambridge Prognostic Group classification, which is a 5-level system predicting clinical PCa specific mortality (22, 23).

Interestingly, the 2012 Briganti nomogram predicting the risk of PLNI has the intrinsic pattern of integrating several clinical factors in a final risk score that associates with adverse pathology in the surgical specimen (12); as such, it has the potential for predicting the natural history of PCa. We tested this hypothesis in the present study, focusing on a very special subset of IR PCa patients with PSA <10 ng/mL, ISUP grade group 3, and clinical stage up to cT2b, a frequently situation in clinical practice, which needs active treatments when life expectancy is at least 10 years.

Our results demonstrated that the 2012 Briganti nomogram not only associated with adverse pathology in the surgical specimen, but also with disease progression; accordingly, as the risk score increased, so patients were more likely to have tumor upgrading, and to experience PCa progression. Interestingly, the nomogram predicted the subset of upgraded and not upgraded patients who progressed; indeed, as the risk score increased, so upgraded and not upgraded patients were more likely to progress; conversely, upgraded and not upgraded patients presenting with lower scores were less likely to progress. As a result, the nomogram was able to stratify progressing patients, independently by the occurrence of adverse pathology in the surgical specimen. These findings are a novelty with the view to manage this subset of patients. Nevertheless, it should be noted that rates of progressing patients belonging to both favorable (≤6%) and unfavorable (>6%) groups were 17.3 and 26.4%, respectively; so, despite the independent predictor status achieved by the nomogram considered either as continuous or a categorical variable, not all patients presenting with a nomogram score >6% will experience progression, and approximately 3 out of four subjects in this category were not identified by the nomogram cut off set at the median.

The category classified as IR is large, heterogenous, and controversial for being not equivalent for the two main systems delivering recommendations for managing the disease (1, 2). Indeed, the NCCN includes patients presenting with intraprostatic bilateral palpable tumors (clinical stage T2c) that are classified as high-risk according to EAU; as a result, the two main classification systems prospect completely different prognostic scenarios. Nevertheless, palpable tumors limited to no more than one half lobe of the prostate (clinical stage T2b), and presenting with PSA <10 ng/mL, as well as with ISUP 3 include a numerous challenging subset of patients, who need active treatments, when life expectancy is at least ten years for both systems. Therefore, assessing prognostic factors for this subgroup of IR patients will be important for both systems, as well as in clinical practice. Our findings bring important information. We showed that the 2012 Briganti nomogram categorized according to the median was able to identify prognostic risk categories. Indeed, tumor upgrading in the surgical specimen was an unfavorable event for the risk of PCa progression; however, not all upgraded patients experienced disease progression; conversely, a subset of not upgrade patients were still likely to recur. Accordingly, we demonstrated that the preoperative 2012 Briganti nomogram was able to identify patients who were more likely to progress, independently by the occurrence of favorable or unfavorable tumor grade in the surgical specimen. As a result, this information might be important for counselling this subcategory of patients for both urologist and radiation oncologist. As recommended, active treatments are considered when life expectancy exceeds 10 years; conversely, when life expectancy does not exceed 10 years, patients presenting with a risk score up to 6% may be safely monitored while those with a risk score above 6% need surveillance in order to detect and treat disease progression without delaying treatment. Taken together, the results of the current study bring the message that the 2012 Briganti nomogram, as well as other potential tools available in predicting adverse pathology, despite their intrinsic limitations, should always be interpreted as potential predictors of adverse cancer control outcomes too, especially when values exceeded the standard cut-off.

Several limitations should be addressed. First, this is a retrospective study and shares limitations with all similar studies, which relied on retrospective data. Second, mpMRI findings were not assessed for not being available in all cases; therefore, we did not use the updated version of the nomogram, which specifically accounts for clinical stage and Gleason Grade Group based on MRI data, as well as for maximum diameter of the targeted index lesion at MRI, demonstrating higher accuracy compared to other existing tools (24). Third, we did not evaluate the percentage of cancer involving each biopsy core for not being available in all cases. Fourth, RARP and eventually ePLND were performed by several surgeons, thus reflecting real-world practice at tertiary referral centers; however, it is possible that this could have affected the pathological evaluation, impacting on disease progression. Fifth, we defined disease progression as the event of biochemical recurrence and/or local recurrence and/or distant metastases because event numbers prevent us to formally consider these endpoints separately. Finally, median follow up approximates 6 years, which is relatively short compared to 10-15 years that represents the ideal follow-up duration to assess cancer control outcomes in patients with low or intermediate risk of recurrence.

## CONCLUSIONS

In the IR PCa population presenting with PSA <10 ng/mL, ISUP grade group 3 and clinical stage up to cT2b, the 2012 Briganti nomogram represents an independent predictor of disease progression after predicting tumor upgrading too. Accordingly, as the nomogram risk score increased, so patients were more likely to experience disease progression; conversely as the nomogram risk score decreased, so patients were less likely to progress. In this challenging subset of patients, the 2012 Briganti nomogram was able to identify prognostic subgroups, independently by upgrading issues. These considerations might turn out useful for surgeons to identify subset of patients needing different treatment paradigms or a follow-up differentiation.
